# pir-hsa-216911 inhibit pyroptosis in hepatocellular carcinoma by suppressing TLR4 initiated GSDMD activation

**DOI:** 10.1038/s41420-024-02285-9

**Published:** 2025-01-17

**Authors:** Zhouxiang Liao, Lichao Yang, Xiaojing Cheng, Xuejing Huang, Qi Zhang, Daoqiang Wen, Zhenyu Song, Yasi Li, Sha Wen, Yongfeng Li, Meizhen Ou, Zhangnan Huang, Tianqi Liu, Min He

**Affiliations:** 1 School of Public Health, Guangxi Medical University, Nanning, 530021 China; 2 Laboratory Animal Center of Guangxi Medical University, Nanning, 530021 China; 3 Life Sciences Institute of Guangxi Medical University, Nanning, 530021 China; 4 Department of Hepatobiliary Surgery, The People’s Hospital of Guangxi Zhuang Autonomous Region, Nanning, 530021 China; 5 Department of Public Health Sciences, College of Medicine, Pennsylvania State University, Hershey, PA 17033 USA; 6 Department of Hepatobiliary Surgery, Hospital of Guangxi Jiang Bing, Nanning, 530021 China; 7 Key Laboratory of High-Incidence-Tumor Prevention & Treatment (Guangxi Medical University), Ministry of Education, Nanning, 530021 China; 8 State Key Laboratory of Targeting Oncology, Guangxi Medical University, Nanning, Guangxi 530021 China

**Keywords:** Hepatocellular carcinoma, Hepatocellular carcinoma, Piwi RNAs, Cell death

## Abstract

Hepatocellular carcinoma (HCC) is a global health concern, ranking as the fourth leading cause of cancer-related deaths worldwide. However, the role of piwi-interacting RNAs (piRNAs) in HCC processes has not been extensively explored. Through small RNA sequencing, our study identified a specific piRNA, pir-hsa-216911, which is highly expressed in HCC cells. This overexpression of pir-hsa-216911 promotes HCC cell invasion and inhibits cell death, particularly pyroptosis. Knocking out pir-hsa-216911 led to increased cell pyroptosis activity, resulting in the activation of caspase-1 and GSDMD. Further analysis revealed that pir-hsa-216911 targets and suppresses TLR4, a key gene associated with pyroptosis in HCC. In the Huh7 cell line, pir-hsa-216911 knockout confirmed its role in suppressing the TLR4/NFκB/NLRP3 pathway by silencing TLR4. Knocking out pir-hsa-216911 significantly inhibited the formation of Huh7 xenograft tumor. In HCC patients, pir-hsa-216911 was highly expressed in HCC tumor samples with steatosis, suppressing TLR4 expression and inhibiting GSDMD activation. This study introduces pir-hsa-216911 as a new high-expressing piRNA in HCC, which inhibits pyroptosis by silencing TLR4 to suppress GSDMD activation. These findings have significant implications for HCC molecular subtyping and as a potential target for cancer therapy.

## Introduction

Liver cancer is the sixth most diagnosed cancer and the fourth leading cause of cancer-related deaths worldwide [1]. Hepatocellular carcinoma (HCC) is the most common type of liver cancer, accounting for more than 90% of diagnosed cases [2]. The leading causes of HCC include hepatitis B virus, hepatitis C virus, aflatoxins, alcohol consumption, obesity, and metabolic syndrome [3, 4]. These factors trigger acute and chronic liver diseases, leading to liver injury, inflammation, and abnormal cell death [5, 6]. Various forms of programmed cell death (PCD) are well known to play crucial roles in HCC development. Among them, pyroptosis is a form of PCD discovered recently and has attracted widespread attention due to its close relationship with inflammation [7, 8].

Pyroptosis is inflammatory caspases-induced gasdermin-mediated programmed death that responds to injury, infection, and inflammation [9]. There are two primary forms of pyroptosis: canonical and non-canonical [7]. Non-canonical pyroptosis can be initiated in various ways, while canonical pyroptosis is inflammatory-driven and mediated by NLRP3 inflammasome assembly, leading to caspase-1-dependent GSDMD cleavage and release of IL-1β and IL-18 [10–12]. Pyroptosis is involved in inflammatory-driven acute or chronic liver diseases and fatty liver diseases [13–16]. Pyroptosis plays both anti and pro-cancer roles in cancer [17, 18]. In the tumor microenvironment, while cancer cell pyroptosis tends to cause acute inflammation and cytotoxicity, leading to tumor-growth suppression, pyroptosis of immune cells may cause chronic inflammation, which inhibits killer cell activity and fuels tumor growth [19, 20]. Notability, lipid exposure induces mitochondrial reactive oxygen species (ROS) in fatty hepatocytes. This leads to hepatocellular NLRP3 inflammasome activation and subsequent pyroptosis in steatotic hepatocytes, aggravated lipid accumulation, and impaired insulin sensitivity [21–23]. Pyroptosis is closely associated with HCC prognosis and progression [24–26]. Higher caspase-1 activity can promote canonical pyroptosis, reducing HCC proliferation and invasion in vitro and in vivo, indicating that dysregulation of pyroptosis plays a crucial role in hepatocarcinogenesis [25, 26]. As well as many other cancer-related PCDs, pyroptosis is also known to be regulated by non-coding RNAs [27–29].

Piwi-interacting RNA (piRNAs) is a recently discovered regulatory small RNA [30]. First found in germ cells as a transposon suppressor for genome defense, piRNA plays critical roles in somatic cells through its posttranscriptional and transcriptional gene silencing ability [31, 32]. Binding with the PIWI subfamily argonaute protein, piRNAs can participate in various gene-regulatory processes [33]. piRNA-induced silencing complex (piRISC)-mediated mRNA silencing [34–36]. It is emerging that piRNAs play vital roles in multiple cancers by their regulated nature [37]. piRNAs have not been extensively studied in HCC, primarily due to their structural differences, which make them challenging to sequence using conventional small RNA methods [38–40]. Although a few piRNAs associated with HCC affecting cell viability and invasiveness have been identified, their mechanisms are largely unknown [35, 37].

To extend our knowledge about the participation of piRNA in HCC, our research has found pir-hsa-216911 to be highly expressed in HCC. It is a piRNA that silences TLR4 and suppresses caspases-1-induced GSDMD activation by inhibiting the TLR4/NFκB/NLRP3 signaling pathway. Knockout of pir-hsa-216911 almost entirely inhibited tumor formation in mice. In HCC patients, pir-hsa-216911 was highly expressed in all cases with steatohepatitis. pir-hsa-216911 plays critical roles in HCC pyroptosis by suppressing TLR4 and could be a potential target for HCC molecular subtyping and therapy.

## Results

### Improved small RNA sequencing pipeline provided better piRNA coverage and revealed pir-hsa-216911 highly expressed in various HCC cell lines

**Fig. 1:**
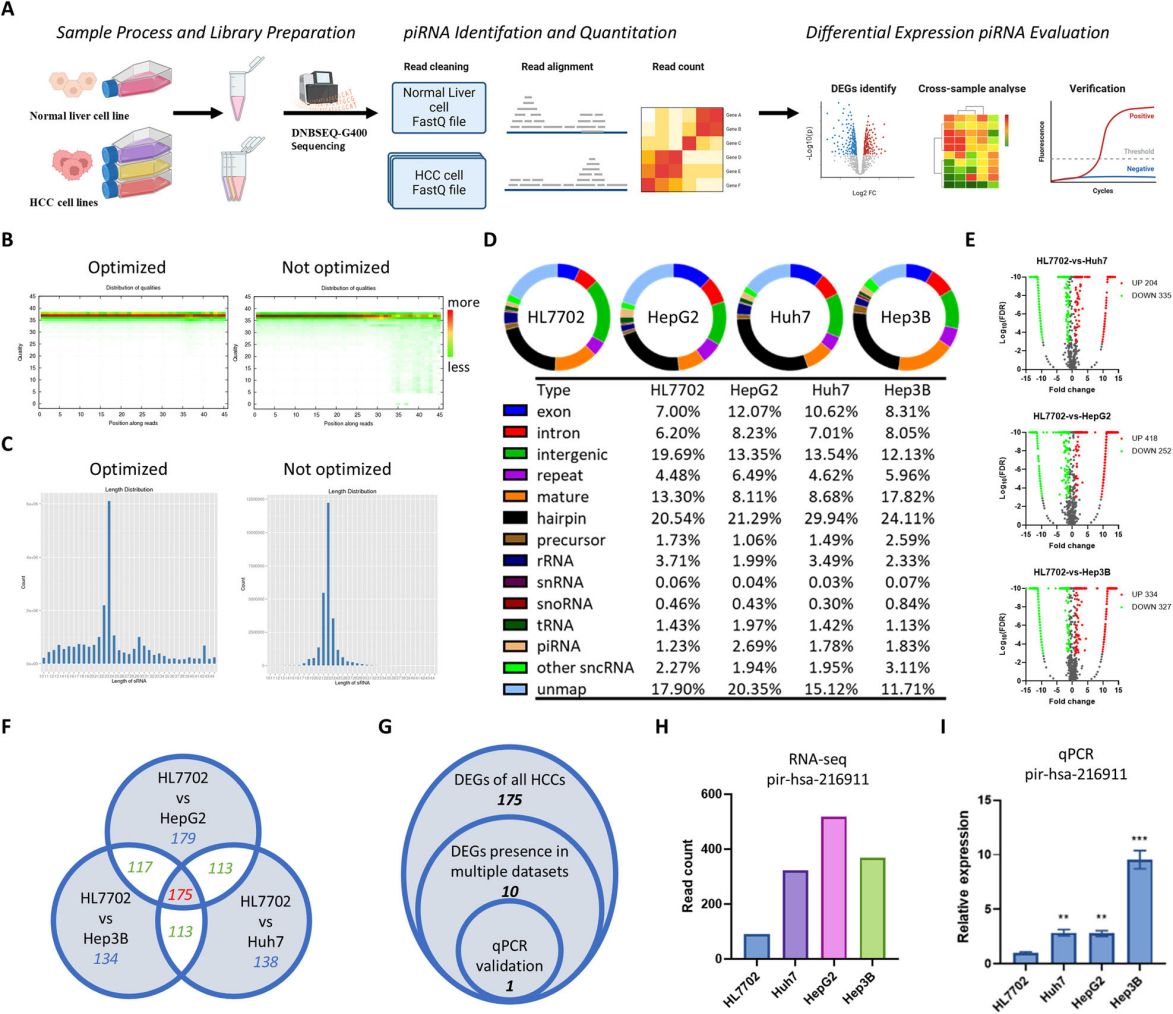
Improved small RNA sequencing revealed that pir-hsa-216911 is highly expressed in various HCC cell lines. **A** The diagram illustrates our small RNA sequencing pipeline. **B** The heatmap displays the quality of reads based on their length. The read quality mainly concentrated at 38, ranging from 1 to 45 nt. **C** This histogram provides a visual representation of the distribution of sRNA tag length. Aside from the primary peak indicating miRNA (19–25), the secondary peak corresponds to piRNA (24–30). **D** The donut plot and list show sRNA tag mapping and annotation results. piRNAs represented only 1–3% of sequenced sRNAs. **E** Volcano plots show the distribution of differentially expressed genes in the three HCC cell lines compared to normal liver cells. **F** The Venn diagram shows the DEG counts in each HCC cell line. **G** The Venn diagram shows the DEG counts in each set. **H** Histogram display reads count distribution in all cell lines in sRNA sequencing result. **I** qPCR showed decreased pir-hsa-216911 expression in HCC cell lines compared to normal liver cells; ***p < 0.001, **p < 0.01 by student-T tests against piRNA expression levels in HL7702.

piRNAs are small RNA that can be analyzed using genomics technology to identify and quantify [30–32]. To minimize individual differences while studying the variation in piRNA expression, three different lines of HCC cells and normal hepatocytes were employed for sequencing (Fig. 1A). As described in the method, we extended the PAGE recovery range to improve piRNA coverage, aiding in improving the identification rate of piRNA. As a result, the read quality of transcriptome sequencing between 27 nt and 45 nt was enhanced (Fig. 1B). The length distribution of reads also showed an enrichment peak in 27nt to 32nt, indicating that the reading quality of sRNAs in the piRNA length range had improved (Fig. 1C). This provided a better basis for subsequent analysis. All sRNA sequencing raw data were available at https://www.ncbi.nlm.nih.gov/geo/query/acc.cgi?acc=GSE215349.

Following the priority described in the methods, 2594, 2828, 3019, and 3140, sRNAs were found in HL7702, Huh7, HepG2, and Hep3B, respectively, among which piRNAs were 241, 242, 255, and 251, respectively. The Piano prediction identified 1025 novel piRNAs within the unmapped tags. As a result, piRNAs represented 1%-3% of sequenced sRNAs, constituting around one-tenth of the miRNA count (Fig. 1D). We identified 1044 differentially expressed piRNAs using a threshold of |fold change | > 1 and FDR < 0.001 (Fig. 1E). Among these, 175 piRNAs exhibited the same trend of expression changes in three types of HCC cells (Fig. 1F).

To narrow down the piRNA candidates, we excluded those only detected in one publication based on piRBase data (http://bigdata.ibp.ac.cn/piRBase/) (Fig. 1G). The remaining ten candidates were validated with qPCR, and only the expression level of pir-hsa-216911 was found to be increased by more than fourfold in various HCC cells (Fig. 1H&1I). Subsequent PIWI-IP assays revealed that pir-hsa-216911 is significantly enriched in the PIWI4 IP product (*p* < 0.001), suggesting it functions as a PIWI4 binding piRNA (Supplementary Figure 1). Considering the consistent expression change trends, bioinformatics search of the piRNA database, and qRT-PCR assay results, pir-hsa-216911 was identified as an HCC-related piRNA candidate.

### pir-hsa-216911 in HCC promotes cell invasion and suppresses cell death

**Fig. 2:**
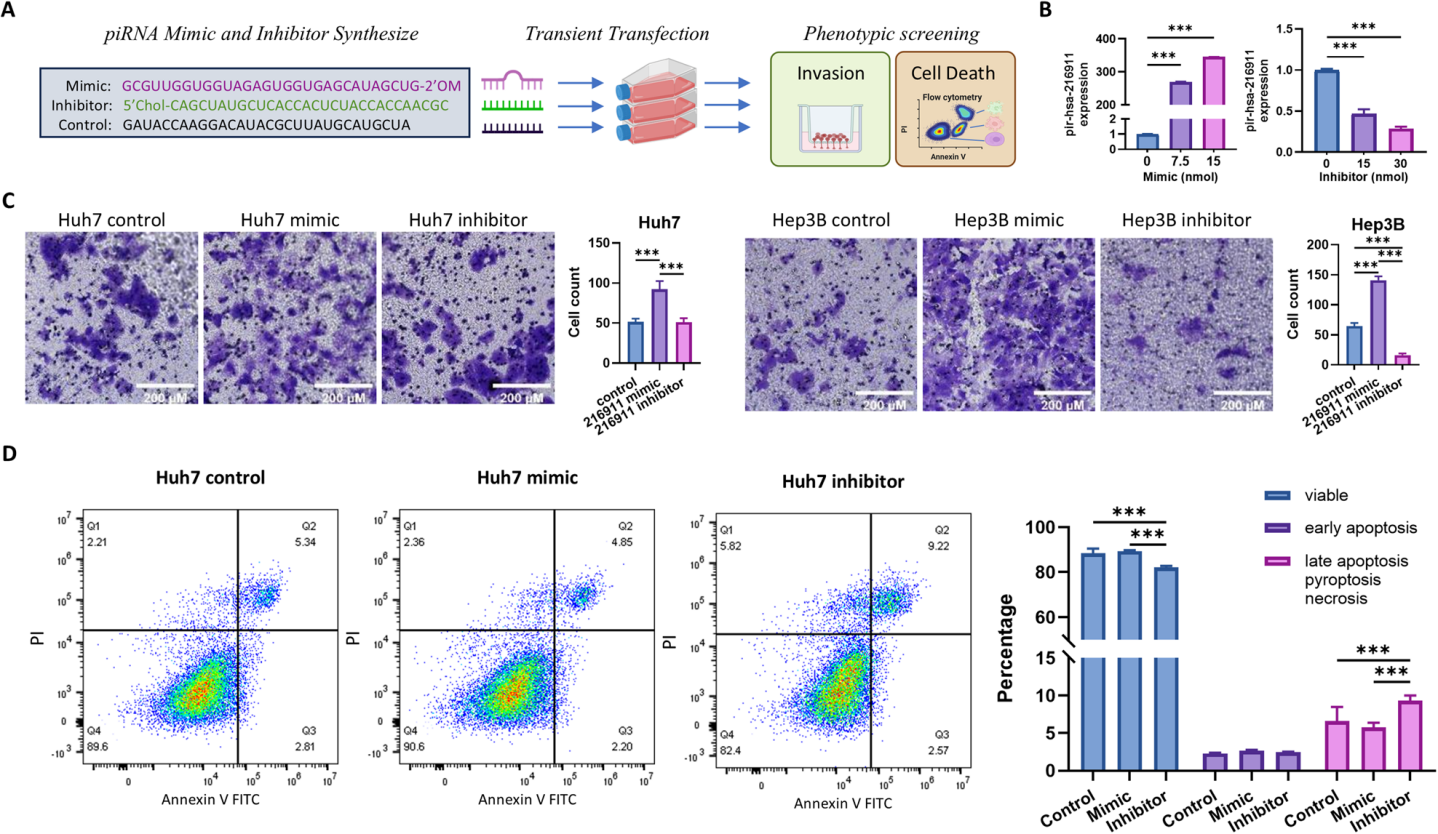
pir-hsa-216911 promotes cell invasion and suppresses pyroptosis-like cell death by transient transfection. **A** The diagram shows the transient transfection design. **B** qPCR confirms pir-hsa-216911 mimics and inhibitors modify the pir-hsa-216911 level in cells. **C** Transient transfection confirms pir-hsa-216911 promotes cell invasion in transwell cell invasion assay. Invasion cells are dyed with crystal violet; histograms represent mean cell counts were statistics under eight visual fields, error bar referee to standard error; ***p < 0.001, ns *p* > 0.05. **D** The flow cytometry assay reveals that pir-hsa-216911 suppresses cell death. The histogram represents three independent repeats. ***p < 0.001, *p < 0.5, ns *p* > 0.05.

It is well known that the abnormal expression of HCC-associate sRNAs usually affects cancer-related characteristics, which can help us reveal its function [41–43]. Based on the piRNA structure, we developed mimics and inhibitors of pir-hsa-216911 (Fig. 2A). The effectiveness of these mimics and inhibitors was confirmed in Huh7, resulting in a 344.03% ± 0.54 fold increase and a 0.715 ± 0.2 fold reduce 48 h after transient transfection, respectively (Fig. 2B). After confirming effectiveness, transient transfection was used to assess cancer-related phenotypes in vitro. Introducing mimics to enhance the expression of pir-hsa-216911 led to an 82.6% increase in Huh7 cells and a 125.8% increase in invasiveness in Hep3B cells. Conversely, when we used inhibitors, a 73.9% decrease in invasiveness was observed in Hep3B cells, but no significant change was seen in Huh7 cells (Fig. 2C). This suggests that pir-hsa-216911 may promote cell invasion in vitro. The flow cytometry assay conducted 48 h after transfection in Huh7 showed that inhibitors of pir-hsa-216911 increased the number of events located in the Anxxin V and PI double positive region by 2.8 ± 0.82% and a 6.3 ± 0.88% reduction of total viable cells. In contrast, mimic transfection didn’t show any significant change (Fig. 2D). Anxxin V and PI double positive cells are representative of plasma membrane permeabilization, which occurs in later apoptotic and necrotic and pyroptosis cells [44–46], indicated an increase in the necrosis ratio, suggesting pir-hsa-216911 was involved in PCD.

**Fig. 3:**
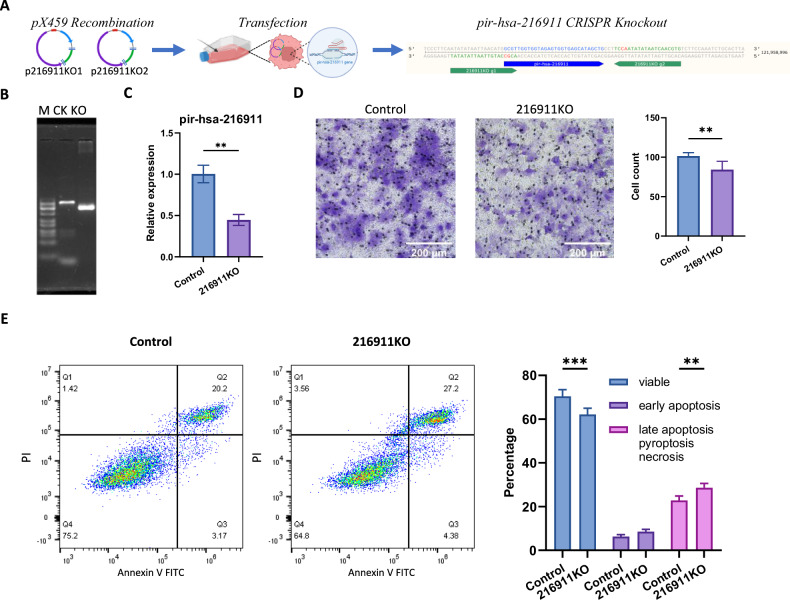
pir-hsa-216911 knockout results in cell invasion suppression and higher cell death in Huh7. **A** The diagram shows the pir-hsa-216911 knockout design. **B** PCR-FLP analysis confirms pir-hsa-216911 knockout. Amplified by the same primers, the KO lane uses 216911KO cell line total DNA as the template, resulting in a sorter PCR fragment, which is different from the control cell line in the CK lane. **C** qPCR showed decreased pir-hsa-216911 expression in the 216911KO multiclonal cell line. **p < 0.01 by student *T* tests. **D** 216911KO cell line shows decreased invasion in transwell cell invasion assay. Invasion cells are dyed with crystal violet; histograms represent mean cell counts, statistics under eight visual fields, and error bars refer to standard error; **p < 0.01 by student *T* tests. **E** The flow cytometry assay reveals that the 216911KO cell line increased in cell death. The histogram represents three independent repeats. ***p < 0.001.

In order to study the function of pir-hsa-216911 and prepare for the subsequent in vivo study, we created a CRISPR knockout cell line called Huh7 216911KO to establish a stable cell line. We designed this cell line to eliminate the pir-hsa-216911 gene from Huh7 cells (Fig. 3A). After transfecting both p216911KO1 and p216911KO2 into Huh7, we screened out the pir-hsa-216911 knockout cell line using puromycin and identified it by PCR-RFLP, demonstrating a distinct fragment pattern from the control cell line (Fig. 3B). Additionally, qPCR analysis confirmed a 55.7% reduction in overall pir-hsa-216911 expression in the multiclonal cell line, confirming the knockout of pir-hsa-216911 (Fig. 3C). When we compared the Huh7 216911KO cell line to the control cell line, we observed a 17.0 ± 4.8 reduction in invasion (Fig. 3D). Flow cytometry analysis revealed a 5.83 ± 1.73% increase in Anxxin V and PI double positive for the Huh7 216911KO cell line (Fig. 3E).

### pir-hsa-216911 knockout results in pyroptosis-prone forming and pyroptosis active

**Fig. 4:**
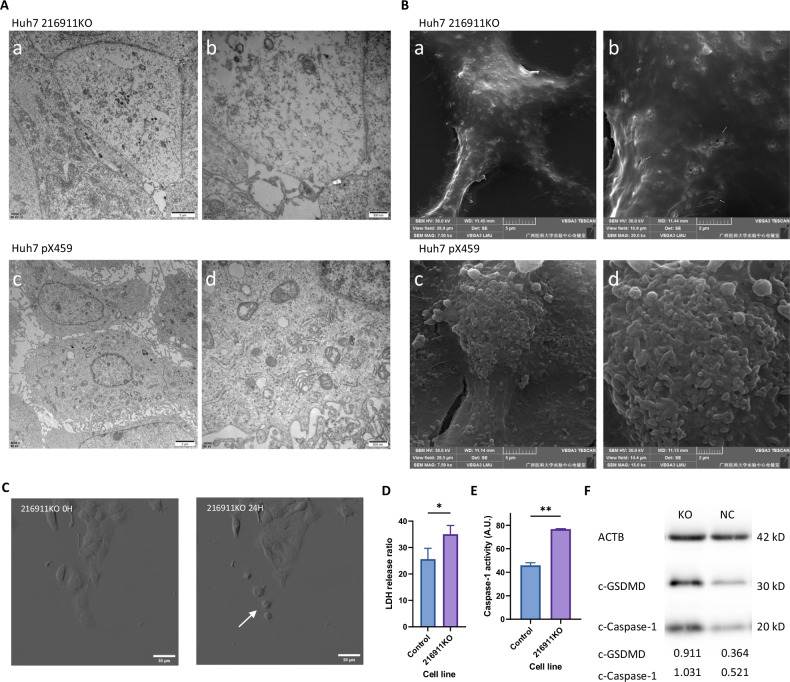
pir-hsa-216911 knockout results in gasdermin pore-forming, increased caspase-1-dependent pyroptosis active. **A** The transmission electron microscope observes cell membrane perforation forming on the 216911KO cell line. The white arrows indicate the perforations on the cell membrane. **B** The scanning electron microscope observes the gasdermin pore-forming on the 216911KO cell membrane. The white arrows indicate typical gasdermin pores. **C** The 216911KO cell line undergoing cell pyroptosis was observed under the optical microscope. **D** The lactate dehydrogenase release assay showed increased cellular pyroptosis in the Huh7 216911KO cell line. *p < 0.05 by student *T* tests. **E** Casepase-1 activity assay revealed that the Huh7 216911KO cell line had a higher caspase-1 activity. A.U. was defined as the amount of enzyme that can cleave 1.0 nmol of the colorimetric substrate Ac-YVAD-pNA in an hour at 37 °C under saturated substrate concentrations. **p < 0.01 by student *T* tests. **F** Western blot displays the 216911KO cell line with higher cleaved-Caspase-1 and cleaved-GSDMD levels than the control cell line.

Each form of cell death has its unique morphology characteristic that can be distinguished [44–46]. Pyroptosis is characterized by forming gasdermin pores, distinguishing it from other forms of necrosis and later apoptosis [7, 9]. In the 216911KO cell line, typical cell membrane perforations were observed using the transmission electron microscope (Fig. 4A). Gasdermin pores were also detected in the 216911KO cell line using the scanning electron microscope (Fig. 4B). Optical microscopy revealed the typical process of pyroptosis in the Huh7 216911KO cell culture (Fig. 4C). These observations indicated the increase in plasma membrane permeabilization was caused by pyroptosis, which resulted from Huh7 216911KO cell line.

In addition to changes in morphology features, increased cell membrane permeabilization and activation of gasdermin by caspases are typical characteristics of pyroptosis [11, 47]. The assay for lactate dehydrogenase release was used to judge membrane permeabilization, which showed a 26.9 ± 8.8% increase in the Huh7 216911KO cell line (Fig. 4D). The activity of caspase-1, a critical enzyme in pyroptosis, also increased by 67.2 ± 6.2% in the Huh7 216911KO cell line (Fig. 4E). Western blot analysis showed higher levels of cleaved-Caspase-1 and cleaved-GSDMD in the Huh7 216911KO cell line compared to control (Fig. 4F), indicating increased pyroptosis activity. These findings demonstrated that the absence of pir-hsa-216911 in Huh7 caused a remarkable increase in pyroptosis activity, suggesting that pir-hsa-216911 inhibited caspase-1-dependent pyroptosis through high expression in HCC.

### pir-hsa-216911 target key pyroptosis genes TLR4, suppressed TLR4/NFkB/NLRP3 axis

**Fig. 5:**
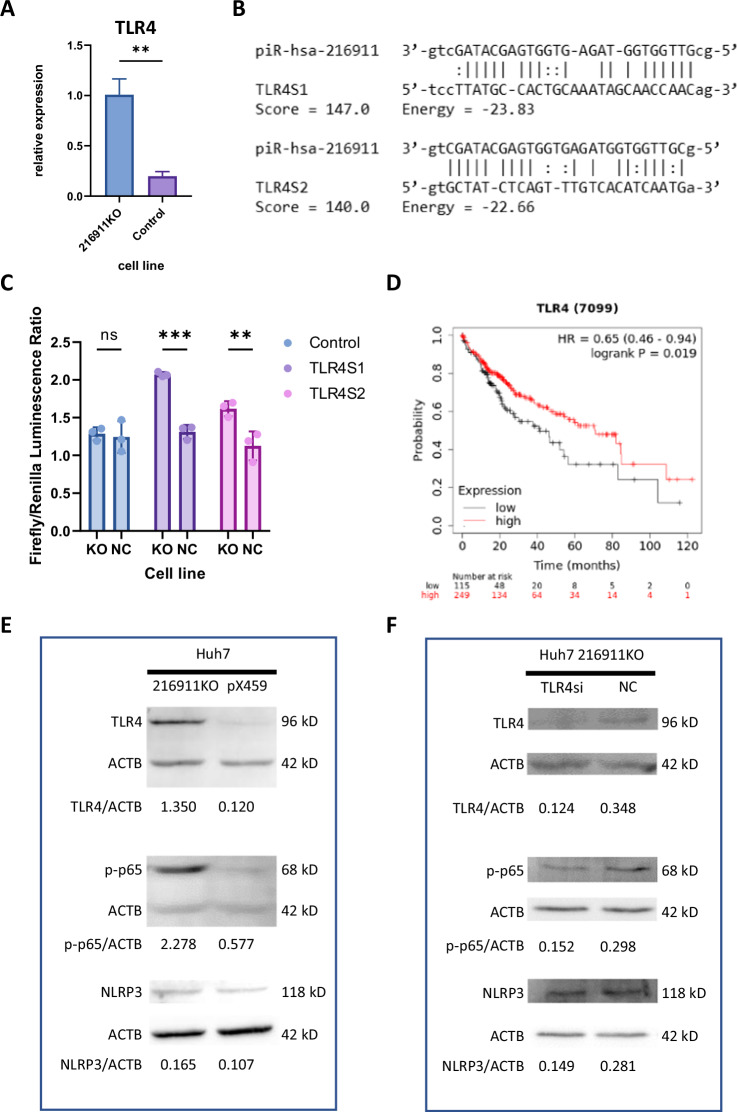
pir-hsa-216911 suppress caspase-1-dependent pyroptosis by suppress TLR4/NFκB/NLRP3 axia. **A** qPCR showed increased TLR4 expression in the 216911KO cell line. **p < 0.01 by student *T* tests. **B** The diagram presented two pir-hsa-216911 binding sites in 3ʹUTR of TLR4. **C** The dual luciferase assay revealed that pir-has-216911 could bind both TLR4 binding sites TLR4S1 and TLR4S2. Plasmids carrying TLR4S1 and TLR4S2 generate a higher Firefly/Renilla Luciferase ratio in the 216911KO cell line, which pir-hsa-216911 was absent. **p < 0.01; ***p < 0.001 by student *T* tests. **D** TCGA-LIHC survival analysis found low expression of TLR4 to be unfavorable to the survival of HCC patients. **E** Western blot analysis revealed pir-hsa-216911 modulating the TLR4/NFκB/NLRP3 signaling pathway. TLR4, p-p65, and NLRP3 levels were elevated in the 216911KO cell line, which pir-hsa-216911 was absent. **F** Western blot analysis revealed pir-hsa-216911 modulating the TLR4/NFκB/NLRP3 signaling pathway through silencing TLR4. After silencing TLR4 with siRNA, pir-hsa-216911 absent could not elevate p-p65, and NLRP3 levels were elevated in the 216911KO cell line.

piRNAs can regulate gene expression by silencing mRNA with piRISC, guided by piRNA-mRNA base complementarity [34, 48, 49]. Using Miranda 3.2, we predicted the targets of pir-hsa-216911 in the human genome and found 10,496 target genes. Among these, we identified 24 genes closely associated with pyroptosis. mRNA sequencing expression profiles indicated downregulations of TLR4, TNFRSF10C, ELANE, and CASP9 in Huh7, HepG2, and Hep3B, compared to HL7702, likely due to high pir-hsa-216911 expression. Among these, TLR4 was significantly increased by 408% ± 82.4 in the 216911KO cell line compared to the Huh7 control (Fig. 5A). TLR4 is the critical sensor of caspase-1-dependent pyroptosis. Within the TLR4 mRNA 3ʹUTR, Miranda predicted two binding sites of pir-hsa-216911-- TLR4S1 and TLR4S2. (Fig. 5B). A dual luciferase assay was conducted to evaluate these predicted binding sites. In comparison to the KO cell line, which lacks pir-hsa-216911, the control cell line exhibited a decrease of 36.8% and 30.4% in the normalized luciferase ratio when carrying a reporter plasmid containing TLR4S1 and TLR4S2, respectively (Fig. 5C). This suggests that pir-hsa-216911 was able to bind to both sites and silent TLR4.

TLR4 is an extracellular sensor for pathogen-associated molecular patterns and is the most well-known pyroptosis sensor [50]. TLR4 expression increases with advancing cirrhosis and promotes HCC but is relatively low in HCC compared to adjacent normal tissue. This reveals critical dysregulation of the pyroptosis gene closely related to HCC [51–53]. TCGA-LIHC survival analysis has found that low expression of TLR4 is unfavorable for the survival of HCC patients, which is consistent with the high level of expression of pir-hsa-216911 in the HCC cell line upon inhibition of TLR4 as expected (Fig. 5D).

Since we have demonstrated that pir-hsa-216911 knockout results in canonical pyroptosis, and TLR4 is well-known to be involved in NLRP3 inflammasome activation, which plays a crucial role in canonical pyroptosis, we assume that pir-hsa-216911 could inhibit caspase-1-mediated pyroptosis by modulating the TLR4/NFκB/NLRP3 signaling pathway [53, 54]. The protein concentration of phosphorylated p65 (p-p65) and total protein NLRP3 was selected to estimate the pathway signal. In the 216911KO cell line with pir-hsa-216911 absence, TLR4, p-p65, and NLRP3 were all accumulated, compared to the control cell line (Fig. 5E). This indicates that pir-hsa-216911 knockout activated the TLR4/NFκB/NLRP3 signaling pathway. Furthermore, silencing TLR4 in 216911KO cell line intercepted this activation, demonstrating that pir-hsa-216911 mainly regulates the NFκB/NLRP3 signaling through TLR4 (Fig. 5F). Taken together, high expression of pir-hsa-216911 in HCC cells can silence TLR4 by binding to the 3’UTR of its mRNA. This TLR4/NFκB/NLRP3 signaling pathway disturbance suppresses caspase-1-dependent pyroptosis.

### pir-hsa-216911 knockout reduce tumor size and activate TLR4/NFkB/NLRP3 axis in Huh7 xenograft model on nude mouse

**Fig. 6:**
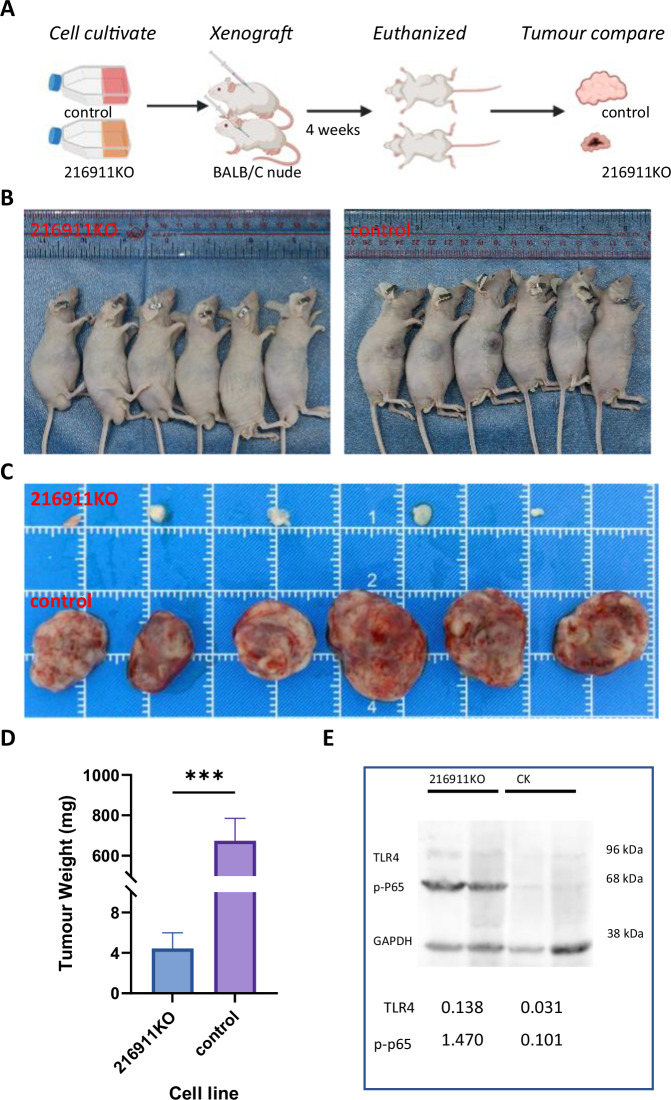
pir-hsa-216911 knockout reduces tumor size and activates both TLR4 and p-p65 in Huh7 xenograft model on BALB/c nude mouse. **A** The diagram presented the xenograft modeling process. **B** Euthanized mouse to display the subcutaneous xenografted tumors. **C** 216911KO group resulted in much smaller tumors in size compared to the control group. **D** 216911KO group resulted in much smaller tumors in weight compared to the control group. The histogram represents the mean tumor weight of each group. ***p < 0.001 by student *T* tests. **E** Western blot analysis revealed pir-hsa-216911 modulating the TLR4/NFκB/NLRP3 signaling pathway in vivo. Both TLR4 and p-p65 levels were elevated in tumors formed in the 216911KO group compared to the control group.

The impact of pir-hsa-216911 on tumor growth was investigated using a nude mouse xenograft model, as depicted in Fig. 6A. After the 28-day experiment, large xenograft tumors appeared near the injection sites of all six mice in the control group, whereas five of the six mice in the 216911KO cell line group developed tumors; most importantly, the volume and weight of the tumors formed by the 216911KO cell line were significantly smaller (Fig. 6C, D). These findings suggest that the absence of pir-hsa-216911 could severely hinder the growth of HCC in vivo. Furthermore, a Western blot assay was used to analyze tumor samples, which showed elevated levels of TLR4 and p-p65 in the 216911KO tumors, indicating the activation of the TLR4/NFκB/NLRP3 signaling pathway (Fig. 6E).

### HCC patients with steatohepatitis highly expressed pir-hsa-216911 in tumor, leads to TLR4 suppression and pyroptosis inhibition

**Fig. 7:**
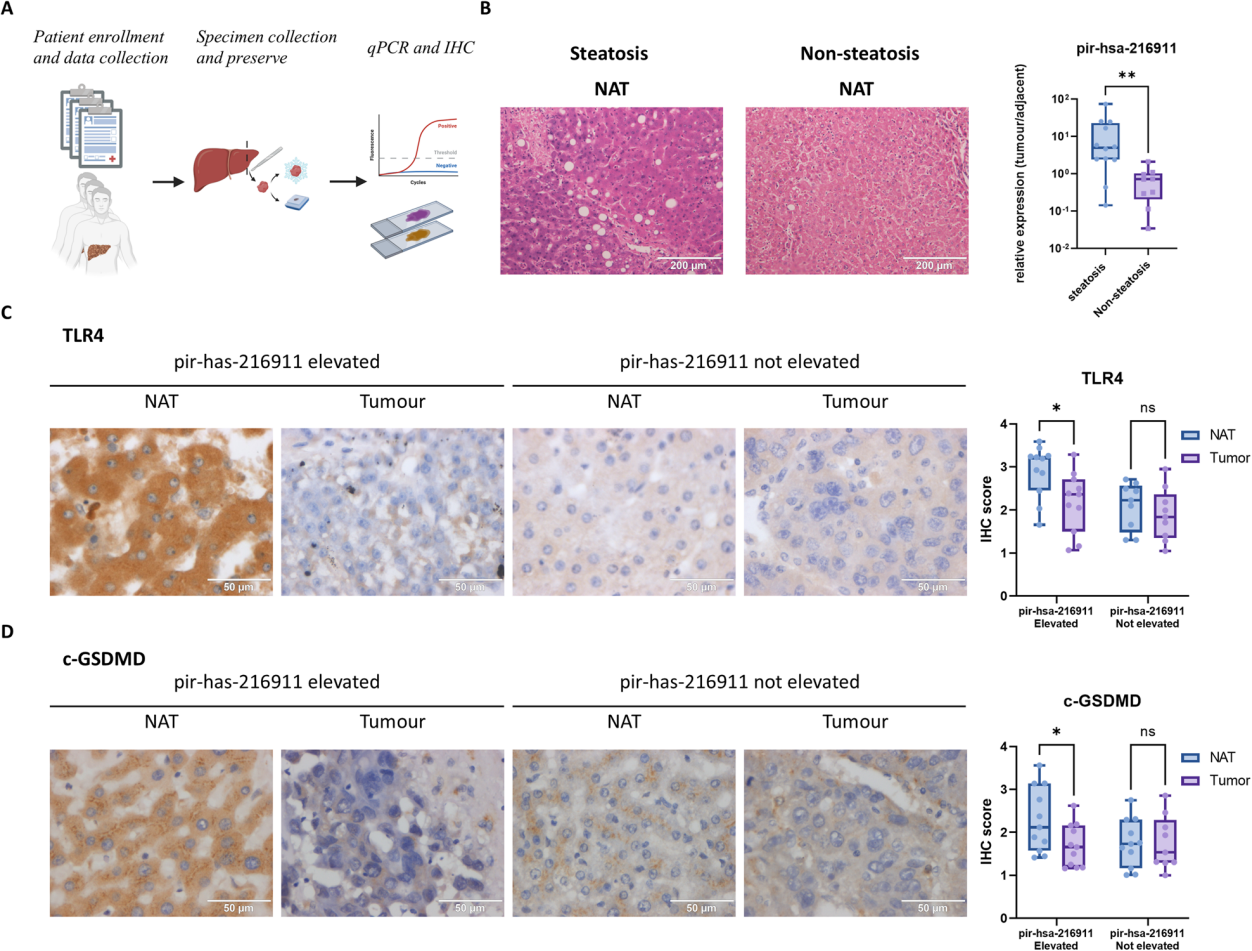
pir-hsa-216911 is highly expressed in HCC clinical tumor tissues with steatohepatitis, suppressing TLR4. **A** The diagram presented the clinical HCC tumor tissue sampling process. **B** The immunohistochemistry demonstrates that TLR4 was downregulated in high pir-hsa-216911 tumor samples. The image shows typical IHC results. The box plot presents all IHC scores. *p < 0.01 by LSD test. **C** The immunohistochemistry demonstrates that c-GSDMD activation was suppressed in high pir-hsa-216911 tumor samples. The image shows typical IHC results. The box plot presents all IHC scores. *p < 0.01 by LSD test. **D** The tumor/adjacent tissue pir-hsa-216911 ratio was higher in HCC patients accompanied by steatosis. **p < 0.01 by student *T* tests.

We conducted a study to investigate the clinical significance of pir-hsa-216911. To validate our findings, we analyzed clinical samples from 20 patients diagnosed with HCC (Fig. 7A and Table 1). Out of the 20 patients, 11 showed more than twofold increase in pir-hsa-216911 expression in their tumor tissues compared to the adjacent normal tissues (NAT), while 9 did not. This suggests that pir-hsa-216911 is upregulated in specific situations within HCC tumors. Further stratification analysis showed elevated levels of pir-hsa-216911 expression in tumors were associated with steatohepatitis (Table 1). The tumor/NAT pir-hsa-216911 ratio was significantly higher in patients with steatohepatitis (Fig. 7B), indicating that pir-hsa-216911 deregulation was correlated with steatohepatitis and fat liver disease.

**Table 1:** Clinical information summary and stratification based on the expression of pir-hsa-216911 in tumors.

Features	*n* = 20	pir-hsa-216911 expression in tumor			
	Cases	Elevated	Not elevated	Coefficient	*P*
Age (years)					
^∞^ > 65	8	5	3	0.123	0.670
≤65	12	6	6		
Gender					
Male	18	10	8	−0.034	1.000
Female	2	1	1		
Edmondson-Steiner grade					
I - II	9	6	3	−0.212	0.406
III - IV	11	5	6		
MVI					
0	7	5	2	−0.363	0.162
1	11	6	5		
2	2	0	2		
Tumor Count					
Single	11	3	7	0.503	0.070
Multiple	9	8	2		
Steatohepatitis					
Steatohepatitis	11	11	0	0.903	<0.000***
No steatohepatitis	9	1	8		

The effect of pir-hsa-216911 on inhibiting pyroptosis was tested using immunohistochemistry. In tumors with high levels of pir-hsa-216911, there was a complete suppression of TLR4 expression in all 11 cases (Table 2), leading to a significantly lower average IHC score (Fig. 7C). Similar trends were observed in the action of GSDMD, with active c-GSDMD showing a high reduction rate in 9 out of 11 cases (Table 2) and low average IHC score in tumors with high pir-hsa-216911 expression (Fig. 7D). These results indicated pir-hsa-216911 acted as a TLR4 suppresser in HCC patients to inhibit pyroptosis.

**Table 2:** Correlation analysis between pir-hsa-216911 elevation, TLR4 expression, and GSDMD activation.

Features	pir-hsa-216911 expression in tumor			
	Elevated	Not elevated	Coefficient	P
TLR4 Tumor/NAT				
Not suppressed	0	6	0.724	0.002**
Suppressed	11	3		
c-GSDMD				
Not suppressed	2	7	0.596	0.022*
Suppressed	9	2		

## Discussions

While various non-coding RNAs can regulate well-known pyroptosis, it is still largely unknown whether piRNA can also regulate pyroptosis [27–29]. A recent study suggests that a single piRNA induces pyroptosis in spermatocyte cells [55]. Still, there is no evidence that piRNA is involved in pyroptosis in somatic cells or cancers. In this study, we identified pir-hsa-216911, a piRNA that suppresses pyroptosis in HCC for the first time, providing a new perspective on the regulation of pyroptosis.

According to our results, pir-hsa-216911 suppresses pyroptosis in HCC by inhibiting the TLR4/NFκB/NLRP3 axis. Although the pir-hsa-216911-TLR4 binding sites didn’t score highly in the miRanda prediction, we noticed a significant increase in TLR4 expression when this piRNA was knocked out. This might be due to the less strict targeting mechanism of piRNA compared to miRNA [48, 49], and the presence of multiple binding sites in TLR4 mRNA amplifying the silencing effect [56, 57] strengthens the pir-hsa-216911-TLR4 interaction. TLR4 is well-known as a sensor for caspase-1-dependent pyroptosis. Studies have shown that TLR4 expression increases with advancing cirrhosis and promotes HCC, but it is relatively low in HCC compared to adjacent normal tissue, indicating its close relationship with HCC [51–53, 58]. However, TLR4 might be involved in pyroptosis through pathways other than TLR4/NFκB/NLRP3 axis activation. siRNA inhibition of TLR4 in 216911KO cell line inhibited the activation of NFκB and NLRP3, suggesting that the TLR4/NFκB/NLRP3 axis is the central axis regulated by pir-hsa-216911.

Our study observed that despite the significant caspase-1 and GSDMD activation increase and LDH leakage, the flow cytometry assays show less than a 10% immediate cell death increase caused by pir-hsa-216911 knockout but still caused a great inhibition in a xenograft model. While strong pyroptosis stimulators like lipopolysaccharide or cisplatin often induce a pyroptosis rate exceeding 30%, mild pyroptosis regulation typically has less impact in vitro but not necessarily less effect in vivo [46, 59, 60]. This points to a considerable difference in the impact of pir-hsa-216911 between the two experimental setups. Although in vitro assay may not fully reveal the function of pir-hsa-216911, as it could not completely simulate either tumor microenvironment or steatohepatitis-induced stress, which pyroptosis in hepatocytes is strongly correlated with, the model we used was a well-established subcutaneous cell line-derived xenograft model, commonly utilized in HCC research [61–63]. While the Balb/c nude mouse we use in this study is T-cell immunodeficiency, the remaining intact innate immune system and NK cells can still involved [64, 65]. Pyroptosis in the xenograft model may cause a more severe significant outcome because it alters the tumor microenvironment. A similar situation was observed in a previous study by Chu et al. in 2016, which showed that TLR4 inhibition led to the more significant inhibition of subcutaneous tumor growth compared to in vitro assays [26]. The substantial impact of pir-hsa-216911 knockout in vivo suggests its potential as a therapeutic target for anticancer therapy.

In our clinical samples assay, pir-hsa-216911 exhibited prominent features highly expressed in all samples with steatohepatitis, indicating its close association with fatty liver disease. This is not surprising because TLR4, the target of pir-hsa-216911, is known to play a crucial role in developing steatosis in liver disease by mediating inflammation and metabolic abnormalities. The strong correlation between elevated pir-hsa-216911 levels and liver steatosis suggests that this piRNA could be helpful in molecular subtyping. Higher pir-hsa-216911 levels in tumors allow HCC to adapt to a condition of hepatic steatosis, which may render interventions aimed at improving fatty liver disease less effective in preventing recurrence.

Research on the effects of piRNAs on HCC has been challenging due to their multiple possible modes of gene regulation. Compared to previous studies that stopped at the phenotypic stage, this study further reveals the regulatory mechanism of piRNAs. Despite the general recognition of the relationships between piRNAs and tumors and cellular pyroptosis and tumors, the regulation of cellular pyroptosis by piRNAs has rarely been reported. Our study demonstrates that pir-hsa-216911 inhibits pyroptosis by suppressing TLR4. However, it is also possible that this piRNA could regulate non-canonical pyroptosis through caspase-3/8 and GSDME, which still need further investigation.

In conclusion, we have identified a new piRNA, pir-hsa-21691, that contributes to the development of HCC by mainly inhibiting pyroptosis. This piRNA is found at high levels in three HCC cell lines, promoting cell invasion and inhibiting pyroptosis. Our experiment revealed that its binding and suppression of TLR4 mRNA inhibited the TLR4/NFκB/NLRP3 signaling pathway and prevented pyroptosis. Deletion of pir-hsa-216911 significantly reduced tumor formation in a mouse subcutaneous xenograft model. In patients with HCC, pir-hsa-216911 was highly expressed in those with steatohepatitis, where it inhibited TLR4 expression and suppressed GSDMD activation. Given its significant impact on tumor formation in vivo and its strong association with fatty liver disease, pir-hsa-216911 may serve as a valuable marker for distinguishing different subtypes of HCC and is promising to become a new therapeutic target for HCC.

## Materials and methods

### Cell lines and cell culture

Human normal hepatic cell line HL7702 and hepatocellular carcinoma cell lines Huh7, HepG2, and Hep3B were obtained from the China Center for Type Culture Collection. The cells were cultured in specific media and conditions: HL7702, Huh7, and HepG2 were cultured in DMEM high glucose medium (Gibco, USA) supplied with 10% FBS (Gibco, New Zealand). Hep3B was cultured in MEM (Gibco, USA) supplied with 10% FBS (Gibco, New Zealand). All cells were maintained at 37°C with a CO_2_ concentration of 5%.

### RNA sequencing

Total RNA from the HL7702 Huh7, HepG2, and Hep3B lines were extracted using TRIzol reagent (Invitrogen, USA). The quality of total RNA was analyzed using the Bioanalyzer 2100 system (Agilent, USA). sRNA libraries were prepared from total RNA for the DNBSEQ-G400 platform (MGI Tech, China) using MGIEasy Small RNA Library Prep Kit (MGI, China), during which the max PAGE recovery range was extended from 30 to 50 nt. Sequencing was performed on DNBSEQ-G400 until each library yielded more than 25 million raw tags, with a read length of 50. All low-quality, invalid, polyA-containing, and short tags were removed, resulting in clean tags aligned with the GRCh38.p12 reference genome, using Bowtie2 and processed with UMI tools to reduce the quantitative bias introduced by PCR. Some small RNA tags may be mapped to more than one category in the annotation information of different RNAs. To make sure every unique small RNA is mapped to only one category, we followed this priority order when annotation: miRbase > pirnabank > snoDB > Rfam > sRNA Predictions (miRDeep2, Piano, siRNA)

### Quantitative PCR (qPCR) analysis of piRNA and mRNAs

All primers used for qPCR were listed in Supplementary Table 1. To analyze the expression level of piRNAs, total RNA extracted from cell lines culture was reverse transcripted by miRNA 1st Strand cDNA Synthesis Kit (MR101, Vazyme, Nanjing, China) using specific stem-loop primers. qPCR was performed on LightCycler 480 (Roche, Basel, Switzerland) by ChamQ SYBR qPCR Master Mix (Q321, Vazyme, Nanjing, China) using specific piRNA primers with the stem-loop universally reverse primer, along with U6 snRNA primers set as an internal reference.

To analyze the expression level of mRNAs, total RNA extracted from cell lines culture was reverse transcripted into cDNA using HiScript III RT SuperMix for qPCR (R323, Vazyme, Nanjing, China). qPCR was then performed using target-specific primers and GAPDH primers as the internal reference.

### PIWI-immunoprecipitation assay

The cell lines were cultured routinely in three replicas until they reached 60% confluence, which was then harvested by trypsinization and centrifugation and resuspended in PBS. Add an equivalent part of 2x nuclear isolation buffer (2.56 M sucrose, 80 mM Tris-HCl pH 7.5, 40 mM MgCl_2_, 8% Triton X-100) and keep on ice for 20 min, then pellet nuclei by centrifugation. Discard the supernatant and resuspend the nuclear pellet in freshly prepared RIP buffer (150 mM KCl, 25 mM Tris pH 7.4, 5 mM EDTA, 0.5 mM DTT, 0.5% NP40, 100 U/mL RNAase inhibitor, 1x Protease inhibitors). Split resuspended nuclei into two fractions for mock and IP. Chromatin was sheared using a dounce homogenizer and pelleted, nuclear membranes and debris were pelleted and discarded by centrifugation, and the supernatant was transferred into a new tube. The antibody was added and bound to the protein of interest and incubated at 4 °C with gentle rotation overnight. Protein A/G beads were added and incubated for 2 h at 4 °C, precipitated, removed supernatant, and resuspended in RIP buffer. Stringently washed the protein A/G bead pellet with RIP buffer five times, then collected the bead and drained and washed. Co-precipitated RNAs were isolated by resuspending beads in TRIzol and extracting them according to the manufacturer’s instructions.

### Transient transfection

To manipulate piRNA levels in cell cultures, pir-hsa-216911 mimic was synthesized as GCGUUGGUGGUAGAGUGGUGAGCAUAGCUG-2’OM, and inhibitor as 5’Chol-CAGCUAUGCUCACCACUCUACCACCAACGC, along with random control GAUACCAAGGACAUACGCUUAUGCAUGCUA (GenSys, China). Synthetic piRNA was delivered into cells using Lipofectamine™ 3000 Transfection Reagent (L3000008, ThermoFisher Scientific, MA, USA) following manufacturer instructions. Total RNA was extracted from the cell culture 48 h after transfection, and then piRNA and target mRNA expression were analyzed by qPCR.

### Transwell cell migration and invasion assay

The Boyden chamber transwell assay was used to assess cell migration and invasion. The cells were cultured routinely until they reached 60% confluence. While migration assay use 24-well Transwell plates (3422, Corning, NY, USA) as it, for invasion assay, 100 μL of 16x diluted Corning Matrigel matrix (356234, Corning, NY, USA) in serum-free DMEM medium was added to the insertion cell and incubated at 37 °C for 2 h to form the gel layer coating. After trypsin digestion, 2 × 10^4^ cells were placed in the upper chamber of the insertion cell with three replicas. The plates were then incubated for 48 h at 37 °C with 5% CO_2_. After incubation, the cells in the upper chamber were removed by swab, fixed with 4% paraformaldehyde in PBS, and stained with 0.1% crystal violet in PBS. The insertion cell was imaged using EVOS Cell Imaging Systems (ThermoFisher Scientific, MA, USA) 20x objective, and the number of cells that penetrated the chambers was counted in 5 fields of vision to assess cell migration and invasion ability.

### Flow cytometry analysis

All flow cytometry assays were conducted on CytoFLEX system (Beckman Coulter, USA). The Annexin V/PI double staining flow assay was performed to detect the proportion of programmed cell death in cells. The cell lines were cultured routinely until they reached 60% confluence in three replicas. Trypsin digestion was performed after washing with PBS, followed by Annexin V/PI double staining at 37°C. Cellular Annexin V (FITC) and PI staining levels were detected and the proportion of cells with each type of staining was counted to evaluate the level of programmed cell death after pir-hsa-216911 knockdown or knockout.

### pir-hsa-216911 knockout

Based on the pir-hsa-216911 location, gRNAs were designed to cleave human chromosome II at positions 121958826 and 121958864 using CRISPR. These gRNAs were then ligated into the pX459 plasmid to achieve p216911KO1 and p216911KO2, respectively. The cell lines were cultured routinely until they reached 60% confluence. Plasmids were extracted and transfected into the cells using Lipofectamine 3000 reagent as per the reagent instructions. After 48 h, the transfection medium was discarded, and transfection-positive clones were screened for one week using DMEM high glucose medium with FBS containing 2 μg/mL puromycin. Subsequently, the complete medium without puromycin culture was resumed and incubated for one to two weeks to bring the coverage back to 60%. The knockout efficiency of the polyclonal cell lines was examined using PCRFLP and qPCR. Polyclones with significant PCRFLP pattern change and reduced pir-hsa-216911 expression levels were selected.

### Electron microscope observation

To prepare cell culture samples for electron microscope observation, the cells were cultured until they reached 60% confluence. Following this, they were trypsinized and resuspended in PBS buffer. The cells were then centrifuged at 100 × *g* and fixed in 3% glutaraldehyde in PBS buffer for 2 h. After that, they were resuspended in PBS three times for 10 min each and centrifuged at 100 × *g* for 5 min to remove the glutaraldehyde. Following removing the supernatant, the cell pellets were stained with 1% osmium tetroxide for 1 h and then washed in PBS for 10 min three times. Then the samples were dehydrated sequentially in ethanol at 50%, 70%, 80%, 90%, and 100% (three times) for 10 min each.

For transmission electron microscope (TEM) observation, use acetone to replace alcohol in dehydrated samples three times, each time for 10 min. Then, infiltrate the samples with epoxy resin using a 3:1, 1:1, and 1:3 acetone to resin ratio for 3 h each. Subsequently, the sample was immersed in 100% epoxy resin overnight and then embedded in epoxy resin with 2% hardener. Polymerization should occur at 35 °C for 12 h, at 45 °C for 12 h, and at 60 °C for 24 h for hardening. Finally, the embedded samples were sectioned and mounted on copper specimen grids for TEM observation.

For scanning electron microscope (SEM) observation, the dehydrated samples were immersed three times in 100% hexamethyl disiloxane for 10 min each to displace alcohol, then vacuum-dried. Afterward, the pellets were attached to the sample holder, and the IB3 ion coater (Eiko Engineering, Japan) was used to coat the specimen with gold for SEM observation.

### Live-cell imaging

Cell pyroptosis was visualized under light microscopy using Celldiscoverer 7 (ZEISS, Baden-Württemberg, Germany). 2 × 10^4^ Huh7 cells were plated in a 24-well dish and incubated routinely for 24 h. Subsequently, the culture medium was exchanged with fresh DMEM supplemented with 10% FBS, and the dish was placed inside the Celldiscoverer observation chamber, maintained at 37°C with a CO_2_ level of 5%. Images were captured every 10 min for two consecutive days.

### Lactate dehydrogenase (LDH) leakage assay

The LDH leakage assay was performed to monitor pyrolysis during cell culturing using the LDH Cytotoxicity Assay Kit with WST-8 (Beyotime, Shanghai, China). For each cell line, six wells in a 96-well plate were designated for the total LDH group and the leakage LDH group, along with a blank well containing no cells. Each well was seeded with 15,000 cells in the complete medium and incubated overnight at 37 °C in a 5% CO2 atmosphere for adherence. After incubation, all wells were washed twice with PBS, and 100 μl of fresh DMEM medium was added before further incubation for 48 h. Following this culture period, total LDH and the leakage LDH activity were measured following the protocol provided by the manufacturer, and the cell death rate was calculated using the formula: (Leakage LDH group - Blank well reading) / (Total LDH Group - Blank well reading) × 100%.

### Caspase 1 activity assay

Caspase 1 activity assay was conducted using the Caspase 1 Activity Assay Kit (Beyotime, Shanghai, China). T25 flasks were seeded for each cell line to achieve 30% confluence and were routinely cultivated until they exceeded 60% in three replicas. Once this was reached, all samples were harvested, and caspase-1 activity was determined by measuring the cleavage of pNA from Ac-YVAD-pNA by caspase-1. The total protein concentration was assessed using the BCA assay. Caspase-1 activity was then calculated as units of activity per milligram of protein.

### Western blot analysis

The cells were cultured routinely until they reached 60% confluence in three replicas, washed with PBS on ice, then lysed by adding RIPA Lysis Buffer (Beyotime, China) containing Protease inhibitor cocktail (Beyotime, China) and subjected to ultrasonication. The BCA protein assay (Beyotime, China) determined the protein concentration. 20 μg of proteins were subjected to SDS-PAGE. The separated protein bands were immunoblotted onto PVDF membranes (Millipore, USA) with TransBlot Turbo semi-dry system (Bio-Rad). The membranes were blocked with 5% non-fat milk powder in TBS buffer, incubated with primary antibodies at 4°C overnight, thoroughly washed with TBS, and then incubated with secondary antibodies for two hours at room temperature. Chemiluminescence signals were developed by Super Signal West Pico PLUS substrate (Thermo Fisher Scientific, USA) and visualized using iBright FL1000 (Thermo Fisher Scientific, USA). The antibodies used in this study were Cleaved Caspase-1 Rabbit mAb (4199, Cell Signaling Technology, USA), Cleaved Gasdermin D Rabbit mAb (1:2000 dilute; 36425 Cell Signaling Technology, USA), anti-TLR4 rabbit polyclonal antibody (1:500 dilute; D121751, Sangon Biotech, China), anti-RELA (Phospho-Ser536) rabbit polyclonal antibody (1:500 dilute; D155006, Sangon Biotech, China), anti-NLRP3 rabbit polyclonal antibody (1:250 dilute; D120143, Sangon Biotech, China), anti-ACTB rabbit polyclonal antibody (1:6000 dilute; D110001, Sangon Biotech, China), anti-GAPDH rabbit polyclonal antibody (1:4000 dilute; D110016, Sangon Biotech, China), and HRP-conjugated goat anti-rabbit IgG (1:6000 dilute; D110058, Sangon Biotech, China).

### Animal experiment

The subcutaneous xenograft model on nude mice was used to evaluate the in vivo function of pir-hsa-216911. Twelve SPF-grade, 5-week-old male Balb/c nude mice were randomly divided into two groups, with six mice per cage. The Huh7 216911KO and Huh7 pX459 control cell lines were cultured routinely, trypsin-digested, and adjusted to a concentration of 2×10^7^ with DMEM medium. The cell suspension was mixed with an equal volume of matrix gel on ice for later use. 100 μL of the cell suspension was injected into the left axilla of each mouse. The mice were kept in SPF and monitored for changes in appearance, behavior, body weight, and subcutaneously injected sites for 30 days after the injection. If the tumor’s maximum diameter exceeds 20 mm or the mouse exhibits significant intolerance, that mouse will be excluded and euthanized. At the end of the 30 days, the mice were euthanized, the tumors were removed and subsequent experiments were performed. All investigators, except for one (ZL), who administered the mouse groups and performed the suspension injections, were blinded to the group allocation. All experimental procedures were approved by the Animal Care & Welfare Committee of Guangxi Medical University.

### Clinical sample assay

Twenty paired HCC tumor specimens and matched normal tissue adjacent to the tumor (NAT) specimens diagnosed between 2023 and 2024 were obtained from tumor surgical resection People’s Hospital of Guangxi Zhuang Autonomous Region. Besides the routine pathology report procedures, Samples for the piRNA assay, along with routine pathology specimens, were collected following surgery. Before pathological specimen fixation, a portion of the tissue was quickly frozen in liquid nitrogen for the piRNA assay. The sample was included in this study after the patient was histologically diagnosed with HCC, and the RNA extracted from the rapidly frozen sample was intact. Cleaved Gasdermin D Rabbit mAb (1:250 dilute; 36425 Cell Signaling Technology, USA) and anti-TLR4 rabbit polyclonal antibody (1:100 dilute; D121751, Sangon Biotech, China) were used for immunohistochemistry. The quantitative immunohistochemistry evaluation was based on five micrographs containing typical tissues, utilizing the IHC Profiler score [66]. This study was approved by the Medical Ethics Committee of People’s Hospital of Guangxi Zhuang Autonomous Region. The patient’s written informed consent was obtained, and the study was conducted in accordance with the Declaration of Helsinki.

### Statistical analysis

Continuous variables are presented as mean ± standard error (SEM). All experiments were performed at least in three independent repeats. Differences between the two groups were analyzed using the Student’s *t* test after the F-test to perform a similar variance check. Multiple group comparisons were performed using two-way ANOVA and Bartlett’s test for variance. A *p* value < 0.05 was considered statistically significant unless otherwise indicated. All statistical analyses and graphics were done with R (version 4.0.3), IBM SPSS Statistics 25, or GraphPad Prism 9.

## Supplementary information

Supplementary Figure 1

Supplementary Table 1

Supplemental Material original blots

## Data Availability

The data presented in this study are available on request from the corresponding author. The sRNA sequencing datasets presented in this study can be found in online repositories. https://www.ncbi.nlm.nih.gov/geo/query/acc.cgi?acc=GSE215349. Clinical data from the TCAG-LIHC project were downloaded from the TCGA Research Network (https://www.cancer.gov/tcga).
