# Supplementary figures and images for: pir-hsa-216911 inhibit pyroptosis in hepatocellular carcinoma by suppressing TLR4 initiated GSDMD activation
Source: Cell Death Discov. 2025 Jan 17;11:11. doi: 10.1038/s41420-024-02285-9 (PMC11742400; doi:10.1038/s41420-024-02285-9)

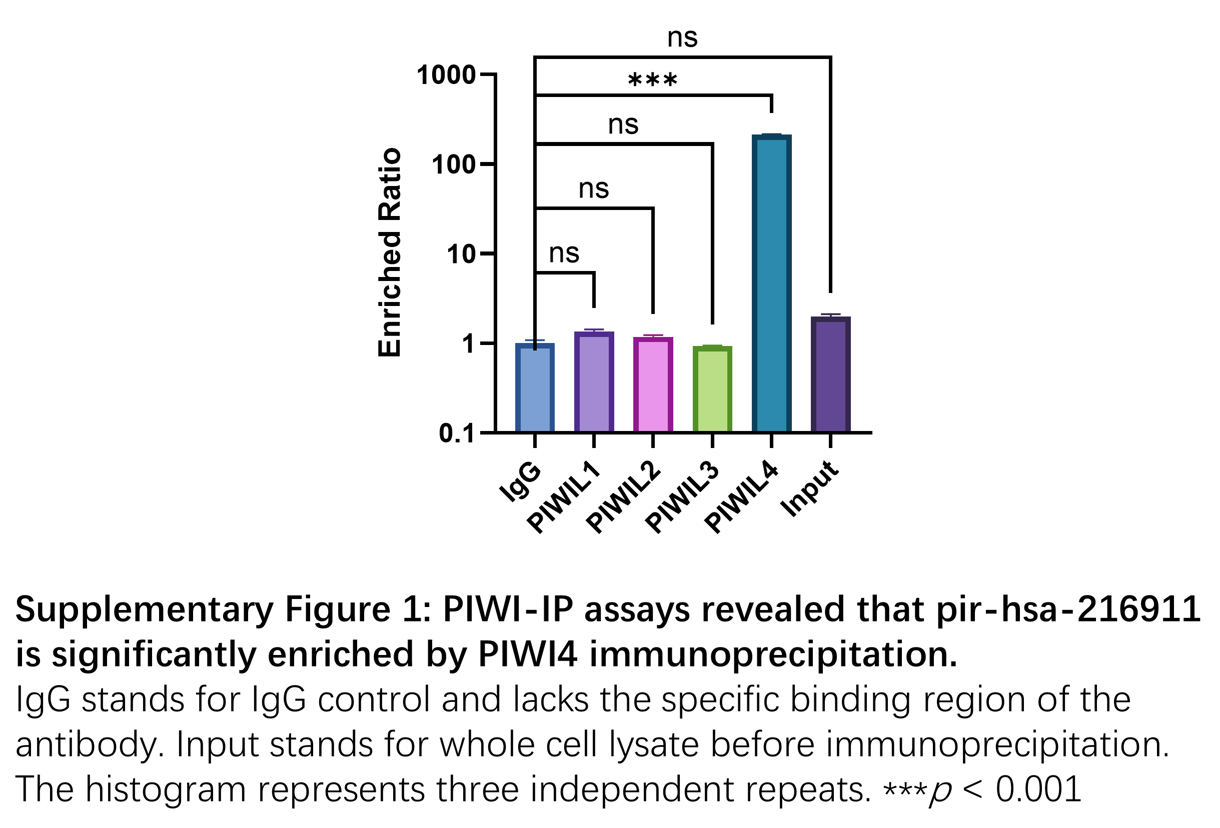

Supplement: Supplementary file 1 — Supplementary Figure 1 [file 41420_2024_2285_MOESM1_ESM.tif]

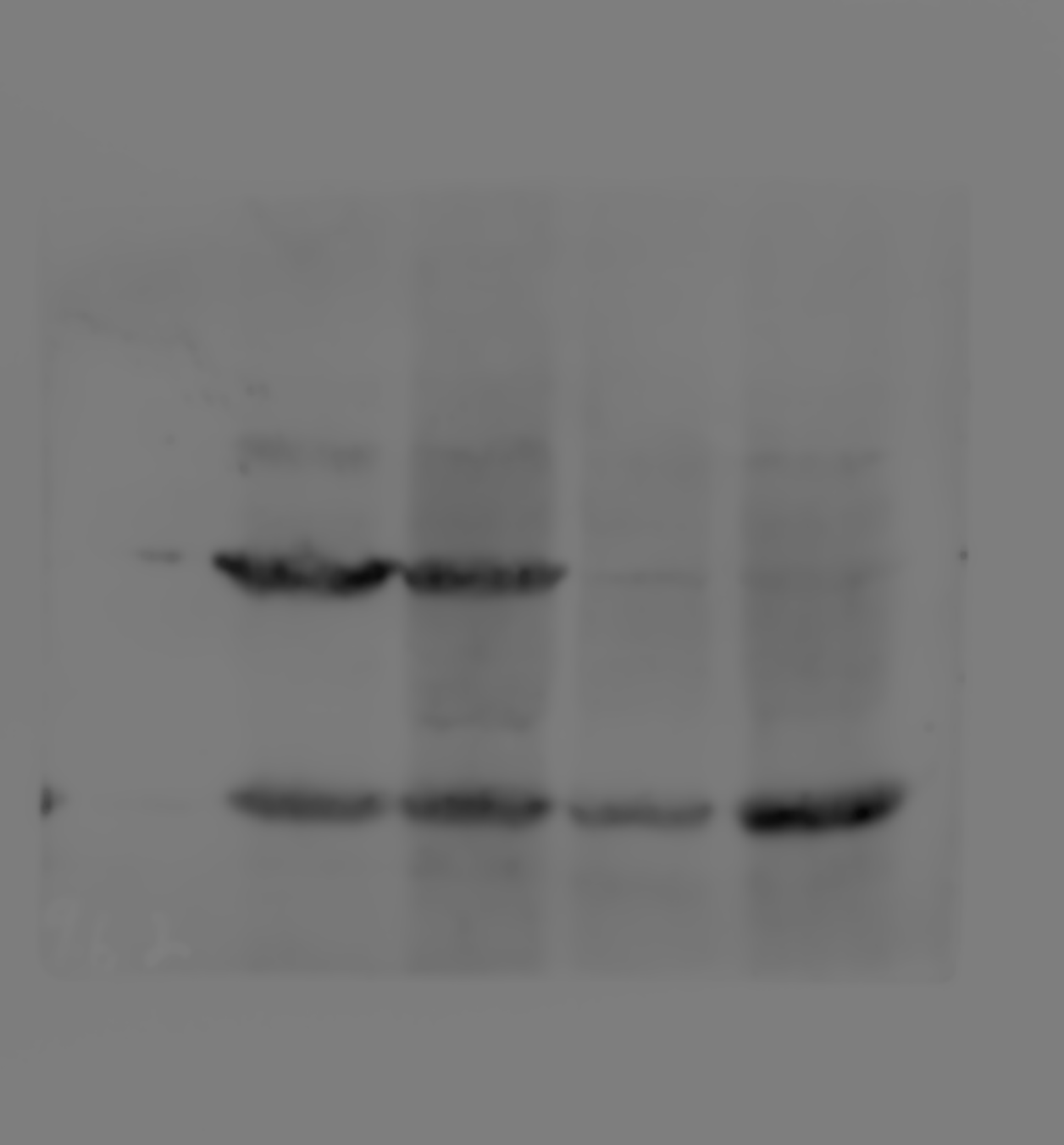

Supplement: Supplementary file 3 — Supplemental Material original blots [file 41420_2024_2285_MOESM3_ESM.zip › Fig6E TLR4 p-p65 load and actual.tif]

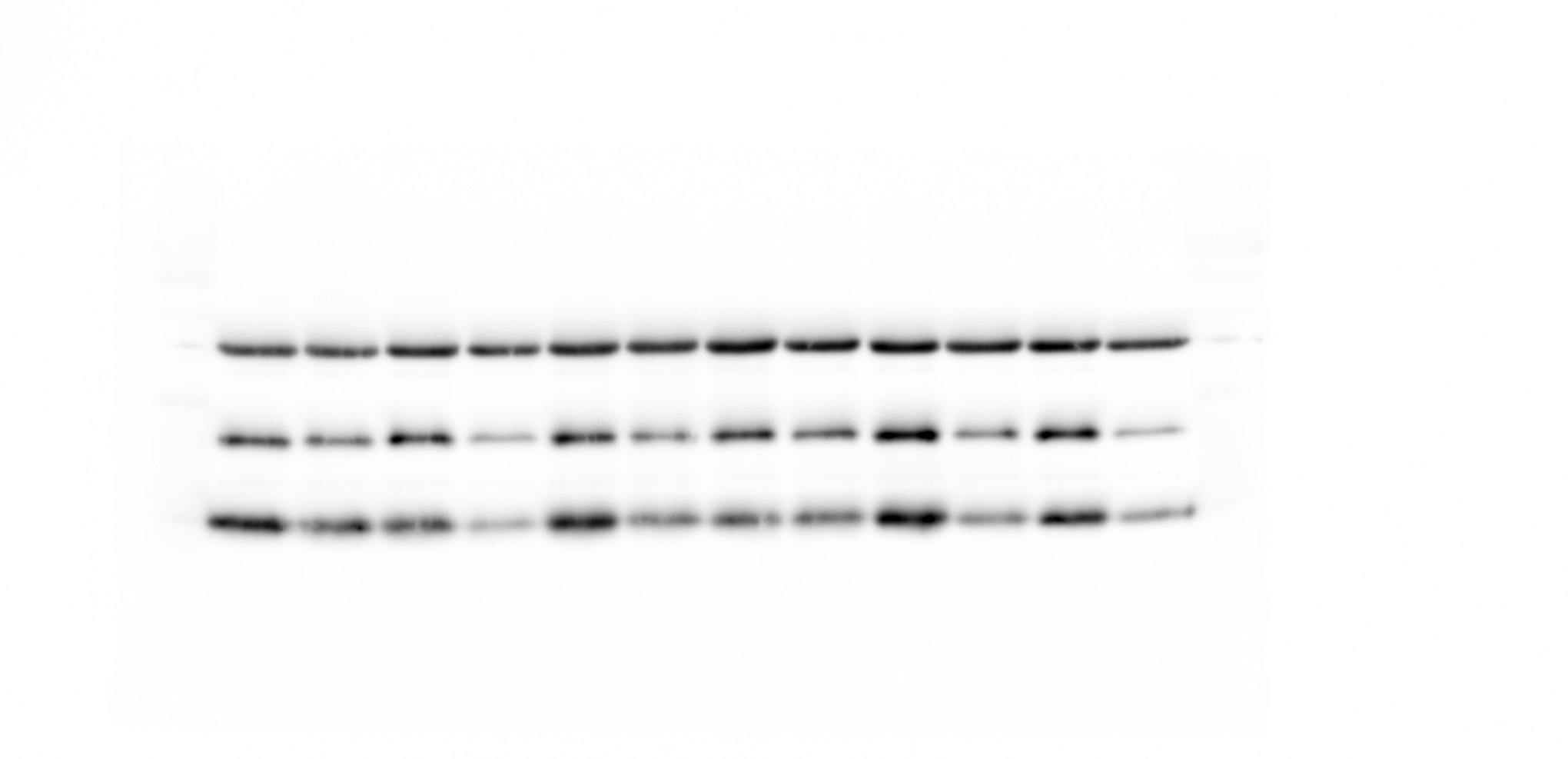

Supplement: Supplementary file 3 — Supplemental Material original blots [file 41420_2024_2285_MOESM3_ESM.zip › Fig4F c-GSDMD CASP1 load and actual.tif]

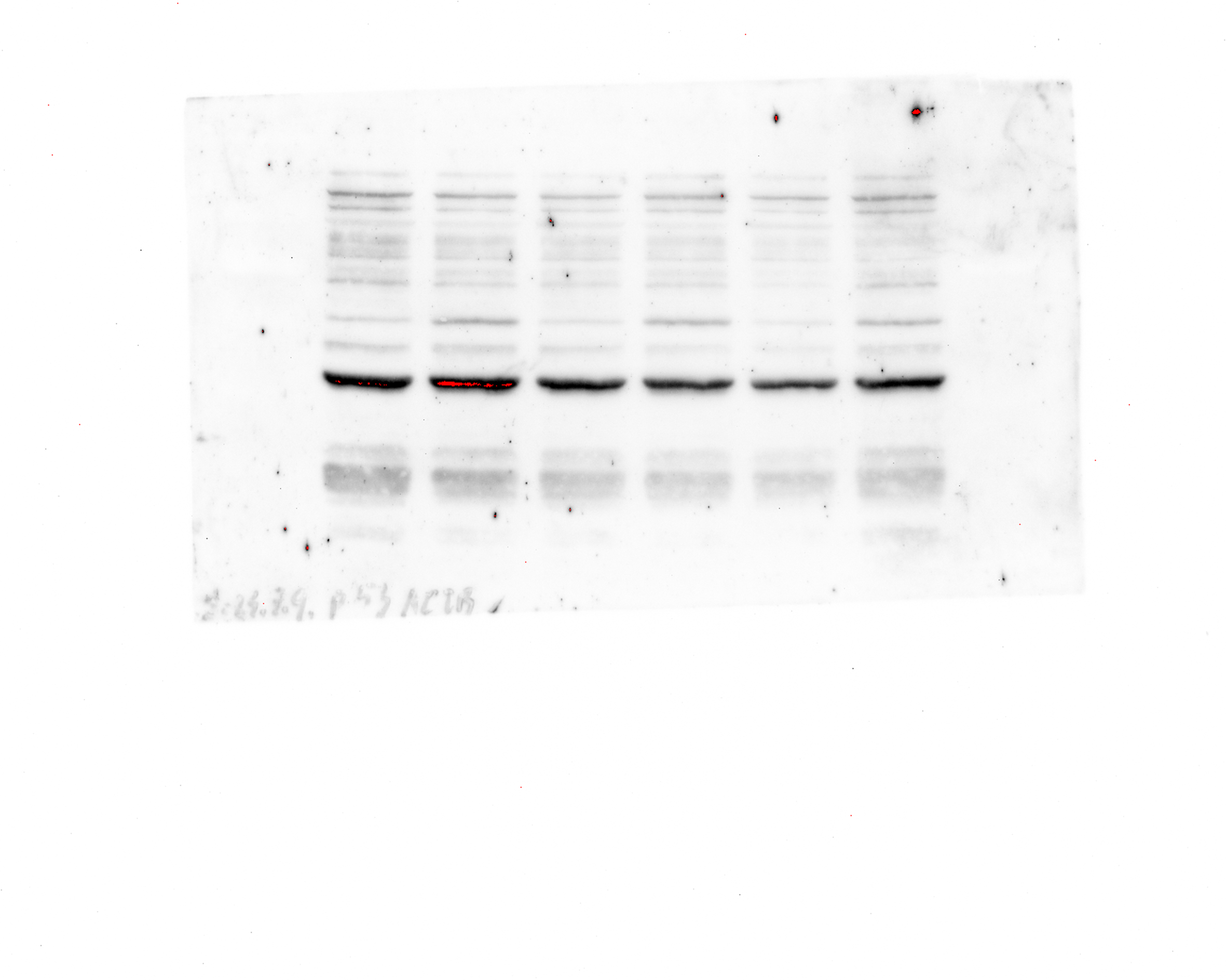

Supplement: Supplementary file 3 — Supplemental Material original blots [file 41420_2024_2285_MOESM3_ESM.zip › Fig5E p-p65 load and actual.tif]

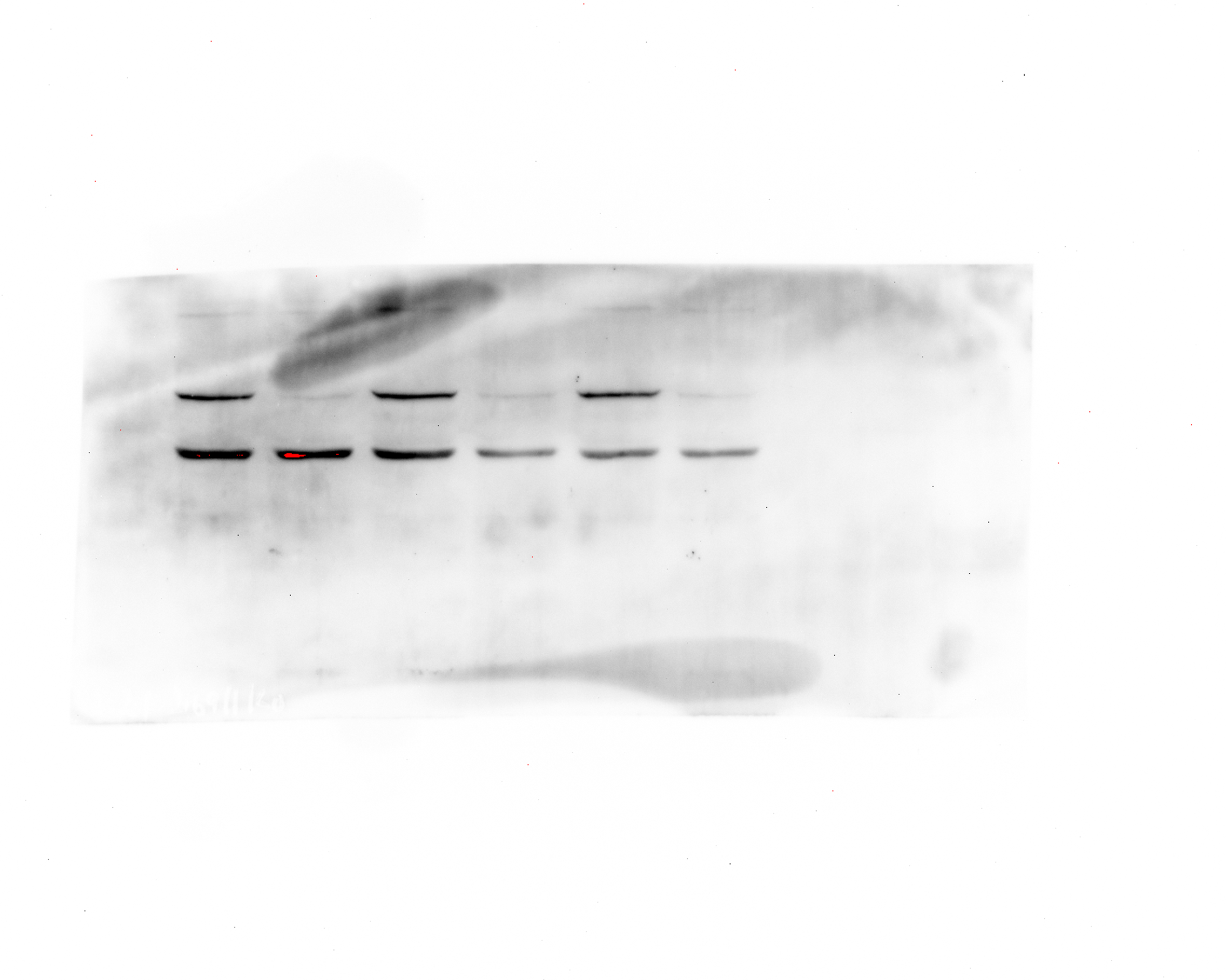

Supplement: Supplementary file 3 — Supplemental Material original blots [file 41420_2024_2285_MOESM3_ESM.zip › Fig5E TLR4 NLRP3 load and actual.tif]

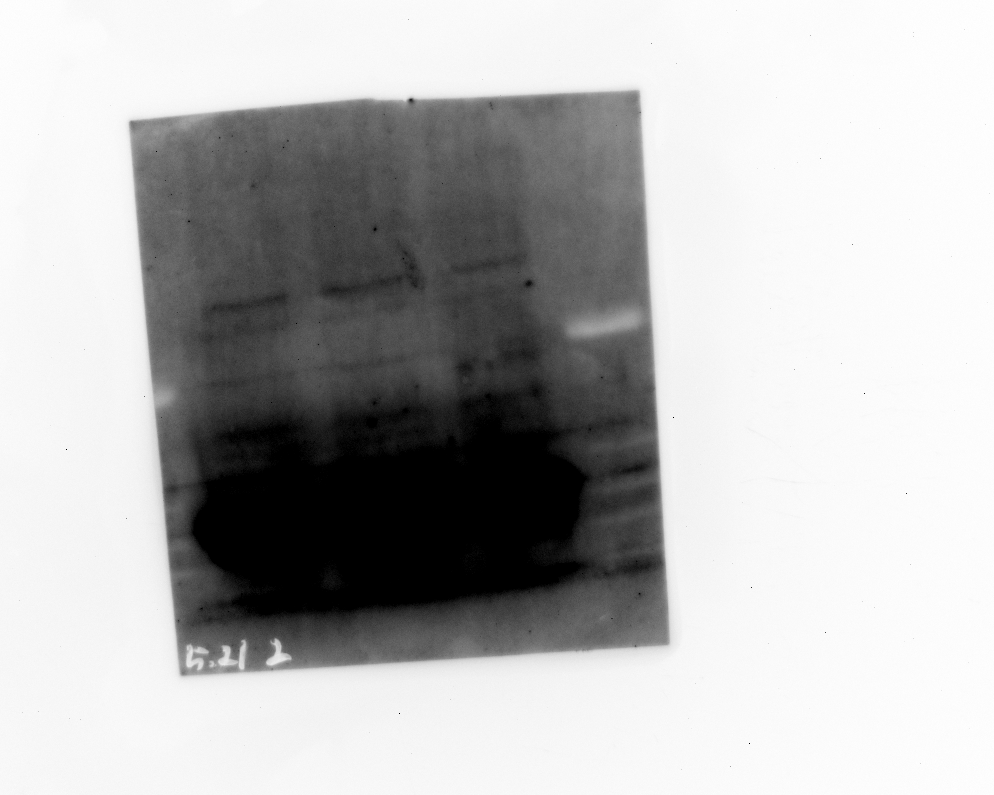

Supplement: Supplementary file 3 — Supplemental Material original blots [file 41420_2024_2285_MOESM3_ESM.zip › Fig5F NLPR3 actual.tif]

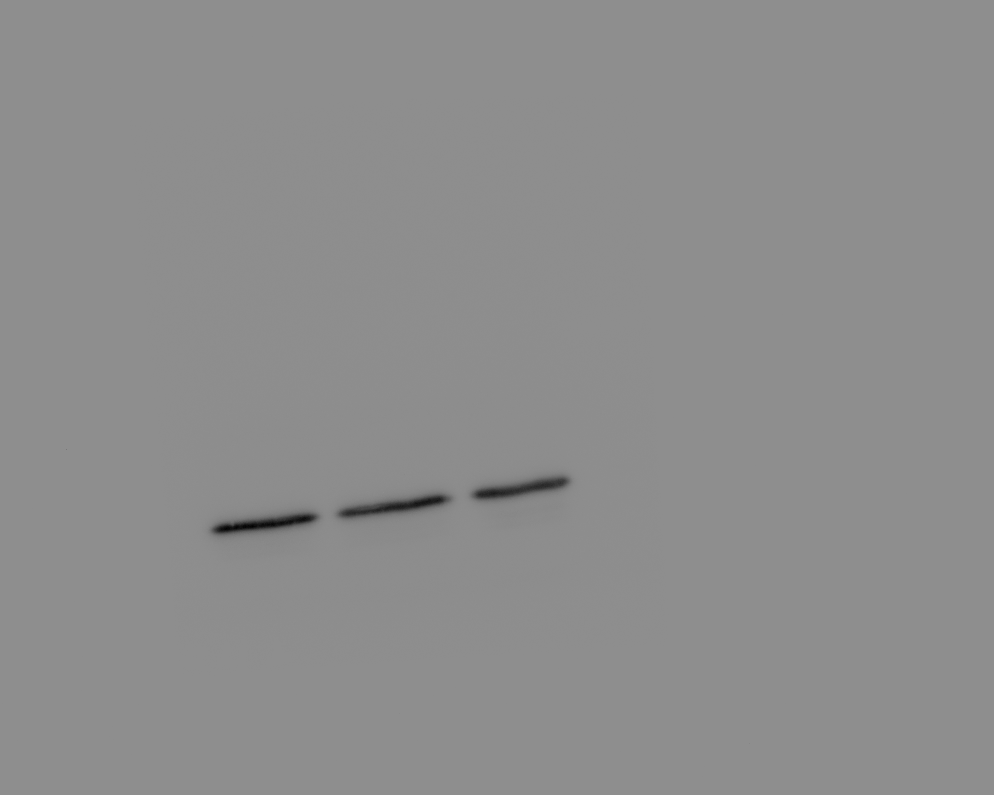

Supplement: Supplementary file 3 — Supplemental Material original blots [file 41420_2024_2285_MOESM3_ESM.zip › Fig5F NLRP3 load.tif]

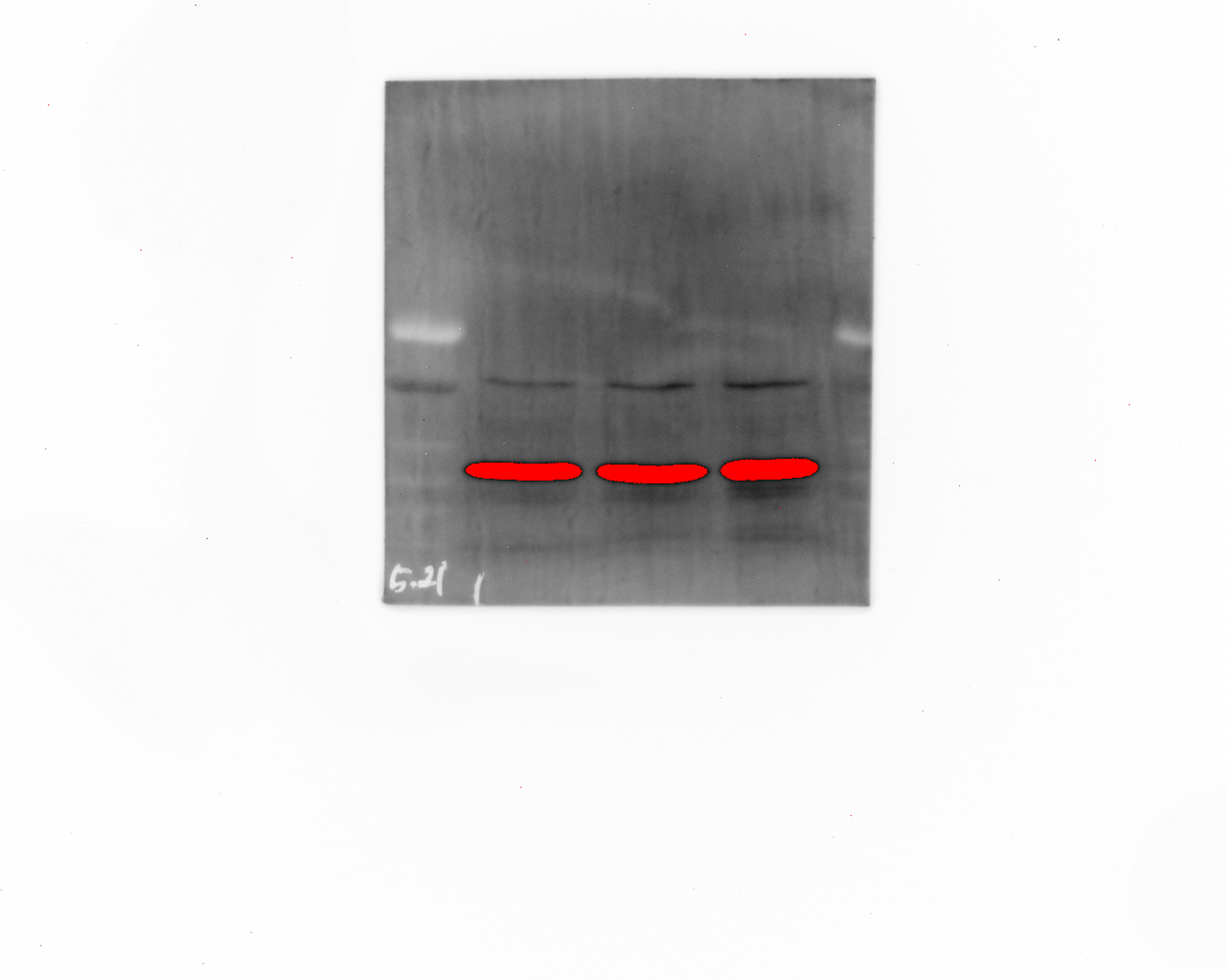

Supplement: Supplementary file 3 — Supplemental Material original blots [file 41420_2024_2285_MOESM3_ESM.zip › Fig5F p-p65 actual.tif]

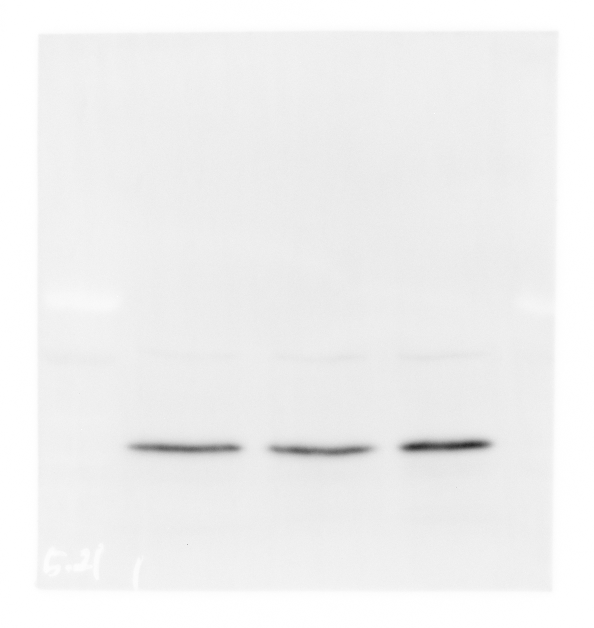

Supplement: Supplementary file 3 — Supplemental Material original blots [file 41420_2024_2285_MOESM3_ESM.zip › Fig5F p-p65 load.tif]

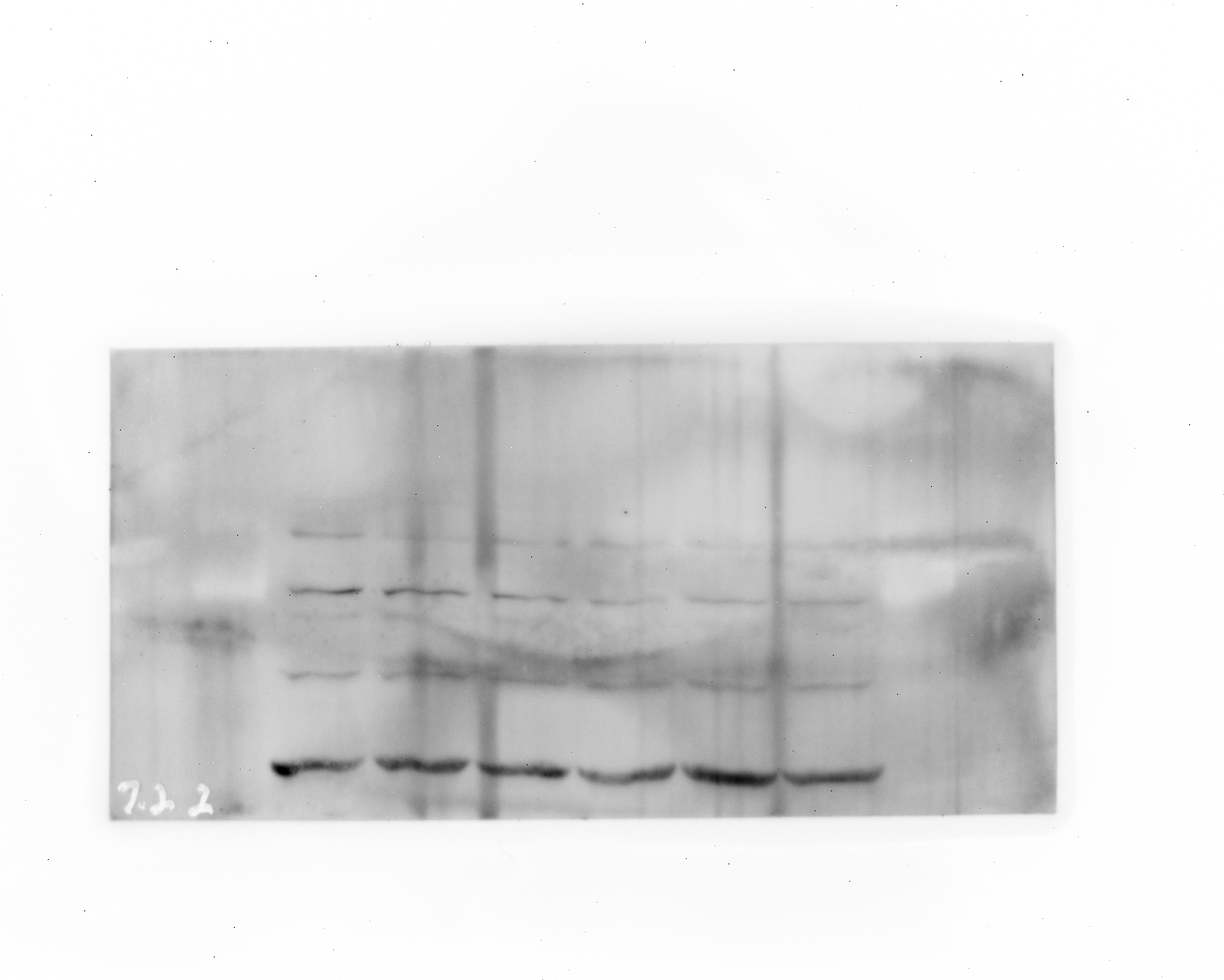

Supplement: Supplementary file 3 — Supplemental Material original blots [file 41420_2024_2285_MOESM3_ESM.zip › Fig5F TLR4 load and actual.tif]
